# Continuous Surveys and Quality Management in Low-Income Countries: A Good Idea

**DOI:** 10.4269/ajtmh.2010.09-0581a

**Published:** 2010-02-05

Dear Sir:

Your publication of the paper by Alex Rowe^1^ is an important contribution to methodological discussions about how best to strengthen and evaluate public health programs in low-income countries. Programs typically have two data sources to monitor, evaluate, and strengthen programs: national cross-sectional surveys and routine information based on their Health Management Information System (HMIS). The cross-sectional surveys are typically population-based, of high quality, and provide a full picture of intervention coverage, illness, and response; but they are expensive and infrequent and usually do not provide local-level results. The routine data collected by HMIS are typically linked to service delivery (e.g., inpatient and outpatient settings), and available locally for review, but the data often are of questionable validity and can seldom, if ever, directly measure intervention coverage or provide full population assessments of illness and response. Rowe's proposed integrated continuous surveys and quality management (I-Q) with small-area analysis could expand the existing HMIS paradigm to produce high-quality programmatically relevant data, while addressing some of the shortcomings of periodic cross-sectional surveys. The approach represents a way forward for governments caught between an increased demand for rigorous results and the need to build sustainable approaches to monitoring and evaluation that produce timely data that can be used to improve programs.

Several positive features explored by Rowe warrant further attention. First, the growing number of initiatives with shared public health aims is a positive development but mean that evaluation designs can no longer rely simply on traditional “gold standard” designs that require non-intervention populations with similar characteristics that can be used as comparison groups.^2^ Revised evaluation designs can build on Rowe's proposed I-Q and analysis approach that can support time series comparisons and dose-response analyses. Second, Rowe's proposal addresses the critical gap between data, quality, and action at national and local levels. The quality improvement process has demonstrated its potential at scale in a few low-income countries,^3^ and Rowe's proposal for quality management structures can help assure that decision making is based on quality data. Third, because data needs may differ between national, regional, and district levels and in different geographic areas, a continuous survey process that can be modified at intervals to adapt to different needs is enticing. Both large national surveys and routine HMIS tend to be rigid and it can be difficult to insert the new questions needed to probe acute or time-limited issues. Rowe's proposed system could allow for a core set of fixed questions and a flexible opportunity to introduce and later withdraw questions as the issues are addressed.

Despite its potential, the practical implications of implementing I-Q and keeping it useful over time will be challenging. A continuous survey system requires a group of full-time employees with a core set of skills in monitoring and evaluation and implies a commitment to information and quality by the staff and by the health leaders of the nation. As Rowe notes, this will require innovative thinking and then sustained buy-in from government officials and donors. By being new, the I-Q approach may be considered risky. If it is simply additional to current intermittent surveys and HMIS, the I-Q approach will have added financial requirements—also risky. If it is meant to supplant existing systems, it will need to prove itself and contend with current advocates for those systems. And, if the approach is championed by a particular Minister of Health or a particular donor health staff who then leaves office, it might be abandoned by a successor.

We believe that Rowe's proposed integrated continuous surveys and quality management can contribute to building national- and district-based systems that combine mechanisms for generating real-time data with broader processes for program decision making. It will be important to obtain a critical mass of supporters to increase the likelihood that a program that might begin as a pilot will be allowed to continue long enough to fully evaluate its utility and costs.

Jennifer Bryce

Institute for International Programs

The Johns Hopkins University

Baltimore, MD

E-mail: jbrycedanby@aol.com

Richard Steketee

Malaria Control Program

PATH

Ferney-Voltaire, France
